# Relative breeding timing and reproductive success of a resident montane bird species

**DOI:** 10.1098/rsos.240769

**Published:** 2024-07-17

**Authors:** Lauren E. Whitenack, Benjamin R. Sonnenberg, Carrie L. Branch, Angela M. Pitera, Joseph F. Welklin, Virginia K. Heinen, Lauren M. Benedict, Vladimir V. Pravosudov

**Affiliations:** ^1^ Department of Biology, Ecology, Evolution and Conservation Biology Graduate Program, University of Nevada, Reno, NV, USA; ^2^ Department of Psychology, University of Western Ontario, London, Canada

**Keywords:** breeding timing, fitness, timing-related constraints

## Abstract

Wild populations appear to synchronize their reproductive phenology based on numerous environmental and ecological factors; yet, there is still individual variation in the timing of reproduction within populations and such variation may be associated with fitness consequences. For example, many studies have documented a seasonal decline in reproductive fitness, but breeding timing may have varying consequences across different environments. Using 11 years of data, we investigated the relationship between relative breeding timing and reproductive success in resident mountain chickadees (*Poecile gambeli*) across two elevational bands in the Sierra Nevada mountains, USA. Chickadees that synchronized breeding with the majority of the population (‘peak’ of breeding) did not have the highest breeding success. Instead, birds that bred early performed best at high elevation, while at low elevation early and peak nests performed similarly. At both elevations, late nests consistently performed the worst. Overall, breeding success decreased with increasing relative timing at both high and low elevations, but the relationship between breeding success and timing differed among years. Our results suggest that in mountain chickadees, earlier breeding is associated with higher reproductive success, especially at high elevations, while late breeding is consistently associated with lower reproductive success at both elevations.

## 1. Introduction

Optimal reproductive timing in animals is likely determined by many environmental and ecological factors, including seasonal patterns of resource availability, weather conditions, predation and competition, and timing of other fitness-related life history events, all of which shape the relationship between breeding phenology and fitness [1–3]. Animals within a population experiencing the same constraints on breeding timing and success may evolve mechanisms to adjust to local conditions (i.e. use of environmental cues) leading to breeding synchrony [4,5]. Despite this prediction, populations show between-individual variation in breeding timing, and individuals that diverge from the majority in their breeding timing may differ in their reproductive fitness as a result. Examining seasonal patterns of reproductive success may reveal potential drivers of these patterns and help forecast demographic changes under future conditions.

It has been proposed that animals may specifically time their reproduction to match the phenology of optimal food abundance, allowing them to maximize reproductive success (i.e. match–mismatch hypothesis) [1,6–10]. However, there are many other factors that can alter the relationship between breeding timing and success. For instance, animals may be constrained by adverse weather conditions especially early in the breeding season, which can lead to decreased reproductive success for early breeders (i.e. environmental constraints hypothesis) [11–14]. In some systems, reproductive success declines across the breeding season as predators become more active [15–17]. Competition for resources within and among species may also affect seasonal patterns of reproductive success, as breeding earlier or later than the majority of the population could benefit individuals by decreasing competition [18,19]. When reproductive phenology dictates the timing of other fitness-related life history events such as migration, food caching or moult, it may place further constraints on reproductive timing [18,19]. For example, some non-migratory bird species residing in alpine environments may experience selective pressure to breed as early as possible, as juveniles that fledge and mature early in the breeding season are more likely to survive the winter and be recruited into the population [20,21]. Furthermore, individuals of higher quality (more experienced individuals or individuals with traits allowing them to succeed under suboptimal conditions, etc.) may be more capable of achieving the benefits of early breeding because they may be better equipped to handle the cost of mismatching with ideal breeding conditions [15,22]. Therefore, several potentially contradicting selective forces may influence animals’ ability to optimize their fitness by breeding at a particular time, and these forces may be highly context specific.

Perhaps unsurprisingly, previous studies investigating the relationships between breeding timing and success across taxa have found mixed results due, in part, to the complex interactions between the aforementioned factors that are not easily deconstructed. Many studies across birds, mammals and invertebrates show declines in reproductive fitness across a given season [23–32]. These seasonal fitness declines have been attributed to various factors including decreased food availability across a season [3,23,32,33], increased predation [15,17,28,32] and constraints related to the timing of development and other life history events combined with the effect of parental quality [20–22,32]. However, in some systems and in certain years associated with harsher weather conditions early in the breeding season, the relationship between reproductive success and timing may be opposite of the commonly observed trend, showing a seasonal increase in reproductive success [11–14].

In addition, some bird species residing in mild temperate climates show an unimodal relationship between breeding timing and success, where individuals that breed closer to peak invertebrate food availability have higher reproductive fitness, including increased fledgling mass (which is associated with post-fledging survival, e.g. [34]) and increased fledgling survival, supporting the match–mismatch hypothesis [35–37]. A recent meta-analysis focusing on European bird species also found evidence for an optimal breeding timing window, but suggested that natural selection is pushing birds to breed earlier, presumably in response to warming spring temperatures that are shifting the timing of the main food resources [38]. However, this relationship is not consistent across bird species [26], likely owing to other factors influencing breeding timing and success. For example, a recent captive experimental study on great tits (*Parus majo*r) created early and late laying selection lines to test the fitness consequences of genetically controlled early versus late breeding timing [39]. Even though birds in the early laying selection line better matched invertebrate phenology, there were no differences in fitness (including survival and lifetime reproductive success) between early and late breeders [39]. The results of this experiment may suggest that factors other than matching food abundance are more important in the wild or that in some systems, differences in breeding timing may not have fitness consequences. In fact, under the ideal free temporal distribution model where animals distribute their phenology across time to account for effects of intra-specific competition, it may be expected that if these individuals do not differ in their quality, they will not experience variation in reproductive fitness based on timing [40,41]. Together, these studies on breeding timing across taxa indicate that the relationship between relative timing and reproductive success is complex, context-specific and likely mediated by many factors.

Much of our understanding of timing-related constraints on breeding in birds comes from lower elevations with mild climates, while less is known about higher montane elevations. In montane regions, which are characterized by more extreme and stochastic environmental conditions, the breeding time window is shorter owing to the earlier onset and longer duration of winter [42,43]. Previous work in alpine systems suggests that breeding early may be particularly advantageous, as juveniles must have sufficient time to mature and prepare for migration or overwintering in a harsh environment [20,21]. In addition, birds that breed early in high-elevation environments may be able to avoid predation as predators are less active early in the spring [16]. However, breeding early may be especially risky at high elevations during some years, as late winter storms and cold snaps can increase nestling mortality via decreased food availability [11–14,44]. Because extreme inter-annual swings in temperature and precipitation are common features of these alpine systems, the relationship between reproductive timing and success may differ across years. Therefore, more research on the reproductive patterns of high-elevation temperate species is necessary to enhance our understanding of optimal reproductive timing across different environmental and ecological contexts.

**Figure 1.**
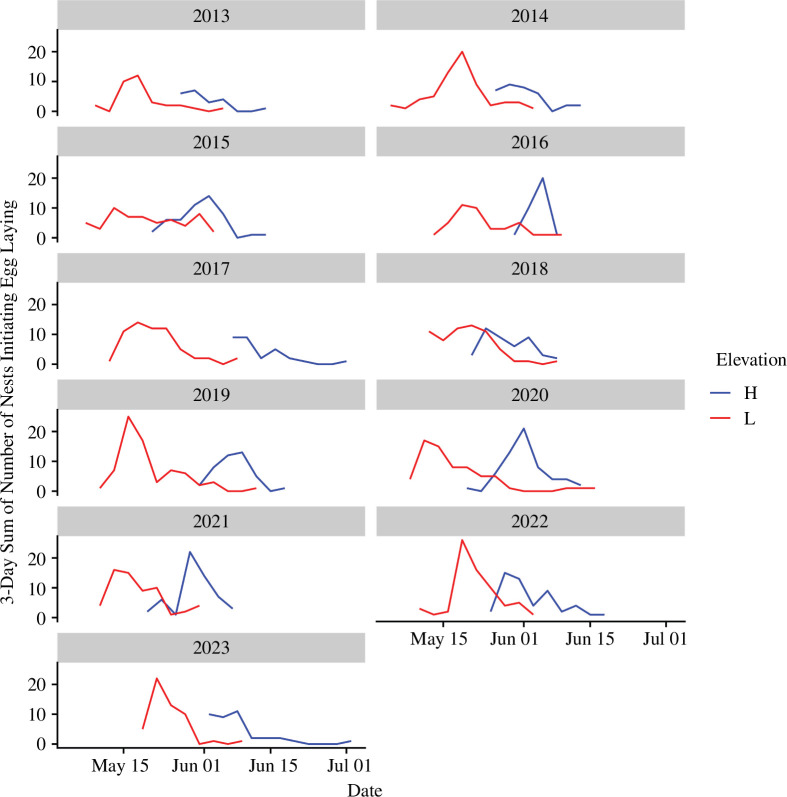
Distributions of first egg dates at low (L) and high (H) elevations across 11 years of breeding data. Three-day sums of the number of nests initiating egg laying were used for smoothing.

We tested whether relative timing of breeding was associated with differences in proxies of reproductive success (clutch size, number of fledglings and fledgling mass) using a long-term mountain chickadee (*Poecile gambeli*) system in the northern Sierra Nevada mountains of North America. Mountain chickadees are non-migratory songbirds that inhabit coniferous forests in western North America and feed invertebrates to young during the breeding season. In montane systems, environmental conditions change rapidly along elevation gradients (e.g. [45]), presenting the opportunity to examine fitness consequences of reproductive timing of a single population under different levels of environmental harshness. We have studied mountain chickadee reproduction in nest boxes for 11 years (2013–2023) at two distinct elevational sites: ‘low’ (range: 1965−2070 m) and ‘high’ (range: 2380−2590 m) [45]. These two elevational bands vary in climate, mostly driven by differences in overwinter snow accumulation (high elevation accumulates much more snow than low elevation, and snow at high elevation persists longer into the summer) [45]. Our previous work showed that at high, but not low elevation, breeding timing was associated with spring snow accumulation (later breeding in years with more snowfall) while yearly variation in spring temperature was not associated with reproductive phenology at either elevation [45]. In this system, there is a distinct peak in the number of nests initiating egg laying within most years, such that the majority of nests are synchronized at both elevations (figure 1), which allows investigation of the potential fitness consequences of such synchronization.

In this study, we specifically tested (i) if there is a fitness advantage to breeding in the ‘peak’ of nesting (breeding more synchronously) across years and between elevations, (ii) whether there are fitness consequences of variation in relative timing of breeding and whether such consequences differ between elevations, (iii) whether the shape (linear or quadratic) of the relationships between relative timing and breeding success (clutch, brood and mean fledgling mass) differ across years and between elevations, and (iv) whether parental age affects breeding timing and its relationship with breeding success.

## 2. Methods

### 2.1. Breeding data

We studied mountain chickadee reproductive biology at the Sagehen Experimental Forest (Sagehen Creek Field Station, University of California Berkeley), California, USA from 2013 to 2023. During the breeding season (April–August) we maintained approximately 350 nest boxes at two elevational sites separated by 3.49 km: ‘low’ (range: 1965−2070 m; coordinates: 39.44350 and −120.243248) and ‘high’ (range: 2380−2590 m; coordinates: 39.42402 and −120.315015) [45,46]. As predation is relatively low in this system and our boxes included anti-predator devices (metal collars below and above the box on trees, metal entrance guards and latches and sheet metal on box lids), it was not possible to investigate predation associated with breeding timing. Therefore, this study focuses on the outcome of parents’ reproductive behaviour: reproductive output measured by clutch size, brood size (the number of fledglings), mean fledgling mass and within-nest variation in fledgling mass. We visited nest boxes weekly starting in mid-April, recorded occupancy status and dates of egg laying. Once we detected nest building, we checked nests at least twice a week until we detected egg laying. Once eggs were detected, we estimated the exact first egg date for each nest based on the number of eggs in the nest (considering that chickadees lay 1 egg per day [47]). From the start of egg laying to incubation initiation, we visited nests every 2 days until we detected incubation start (estimated as the day after the last egg was laid). We then checked the nests for hatching 12 days after incubation initiation. If hatching was not detected, we continued checks every day until hatching occurred. Clutch size was recorded at the onset of incubation. Fledgling body mass and brood size were recorded on day 16 post-hatch (fledging time varies between day 20–24 post-hatch, but day 16 is early enough to prevent force-fledging when processing the young) when all nestlings were weighed to the nearest 0.1 g and banded with a unique numeric aluminium band issued by the United States Geological Survey Bird Banding Laboratory [45,46]. We check nest boxes after birds leave the nest to clean boxes for the next season and we do not typically observe any dead nestlings that were alive at day 16 but died before fledging, so we use the number of young at day 16 (brood size) as a measure of the number of successful fledglings. Mountain chickadees typically raise one brood per season [47] and second broods and renests were extremely uncommon during our study period. Therefore, only initial breeding attempts for each season were used, and renesting or second nesting attempts from the same season were removed (41 renests or second nesting attempts recorded across 11 years). We evaluated variation in fledgling condition by calculating the coefficient of within-nest variation in fledgling mass (CV = (s.d. ÷ mean) × 100). We used the following measures of reproductive output as proxies for reproductive success: clutch size, brood size, mean fledgling mass and CV. Clutch size is highly correlated with brood size or number of fledglings, which represents fledging success. Greater fledgling mass is associated with higher probability of surviving the fledging period [34,48–52]. CV is an indicator of the quality of the nest, as a higher CV suggests that parents invested more in some nestlings compared to others.

Adult birds were banded during the breeding season at nest boxes or using mist nets at established bird feeders at our field site. Each bird was fitted with a unique combination of colour bands and a passive integrated transponder (PIT) tag to track individual reproductive timing and output. The age of birds (first year or older adult) was determined either based on the initial banding date (bird was banded as a nestling) or on molt limits and feather condition as described in Pyle [53]. Sex was determined based on the presence of a brood patch or cloacal protuberance and behaviour, including incubation, as only female chickadees incubate [47]. In subsequent analyses, we categorized birds as either second-year or after second-year birds (i.e. first-time breeders or older breeders). Age data were collected starting in 2015; therefore, analyses including parental age exclude data from 2013 and 2014.

### 2.2. Statistical analysis

We first compared the intra-annual ranges in first egg dates, representing the lengths of the breeding seasons, between elevations (11 ranges for each elevation) using a linear mixed model with range as the dependent variable, elevation as a fixed effect and year as a random effect with the ‘glmmTMB’ package v. 1.1.9 in R with a Gaussian distribution [54].

We analysed reproductive success (clutch size, brood size, mean fledgling mass and CV) as a function of relative breeding timing using two approaches: (i) we separated all nests into three general categories of breeding phenology—early, peak and late, relative within years (‘categorical timing analysis’) and (ii) we used timing of reproduction as a continuous variable relative to the first nest of each season (‘continuous timing analysis’). In our categorical timing analysis, we tested whether there was an overall fitness advantage to breeding synchronously at the peak of nesting. In our continuous timing analysis, we investigated the shape of the relationship between relative timing and breeding parameters, and how this shape differed across years. A threshold of *ɑ* < 0.05 was used to establish significance. All analyses were conducted in R v. 4.3.3 [55].

### 2.3. Categorical timing analysis

**Figure 2.**
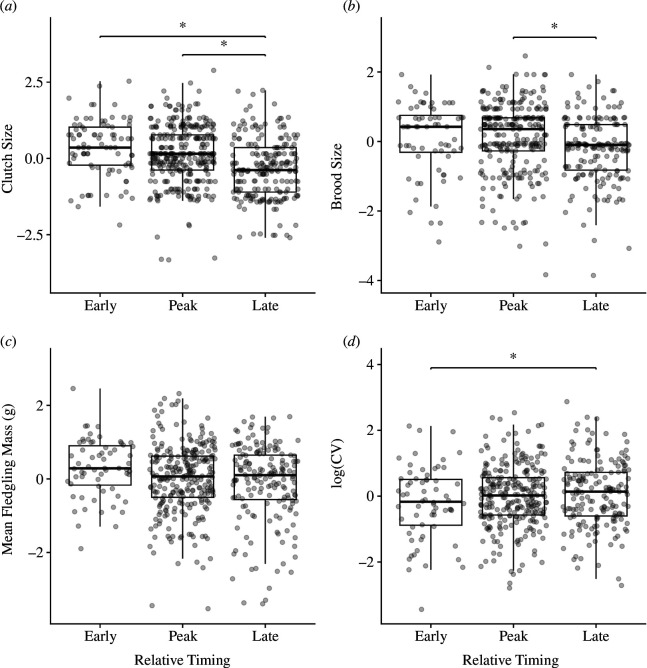
Low elevation**:** relationships between categorical relative timing and breeding variables: (*a*) clutch size; (*b*) brood size; (*c*) mean fledgling mass; (*d*) log-transformed coefficient of variation in mean fledgling mass (CV). Response variables were centred within years for plotting. Asterisks (*) indicate significance of comparison (*ɑ* < 0.05). Data from 2013 to 2023.

For each year of breeding data and at each elevation, we calculated the number of nests initiating egg laying for each 3-day period beginning on the earliest recorded first egg date. We used 3-day periods to identify the shape of the distribution of first egg dates across seasons (used for smoothing; see figure 1). We defined the ‘peak’ in nesting as the two consecutive 3-day periods of nesting that included the highest number of nest initiations (a 6-day period, figure 2). All nests occurring before the peak were categorized as ‘early’ nests and all nests after as ‘late’. High and low elevations were modelled separately as metrics of reproduction vary with elevation (e.g. high-elevation birds start breeding 2–3 weeks after low-elevation birds and generally have larger clutches and broods), and we expected divergent responses across elevations owing to differences in climate [45,46]. We modelled the effect of relative timing (three categories—early, peak and late) on clutch size, brood size, mean fledgling mass and within-nest CV in fledgling mass separately with linear mixed-effects models using the ‘glmmTMB’ package [54], including year as a random effect. Pairwise comparisons between categorical timing factors were calculated using the ‘emmeans’ package v. 1.10.1 with a Tukey adjustment for multiple comparisons [56].

We modelled the effect of relative timing on clutch size and brood size using the generalized Poisson distribution in the ‘glmmTMB’ package [54,57] and modelled mean fledgling mass and within-nest CV in fledgling mass using a Gaussian distribution. Within-nest CV in fledgling mass was log-transformed before running models to improve residual fit. We used the ‘DHARMa’ package v. 0.4.6 to simulate residuals, check the residual fit and check for model misspecification problems [58]. We computed type III analysis of variance (ANOVA) tables using the ‘Anova’ command from the ‘car’ package version 3.1-2 [59].

Parental age has been shown to influence reproductive timing and output, such that experienced individuals usually breed earlier and have increased reproductive output compared with first-time breeders [36,37,60]. We modelled the effect of parental age (i.e. first-time breeders or older breeders, separately for males and females) on categorical relative timing, including an interaction between age and elevation, with ordinal logistic regression using the ‘polr’ function from the ‘MASS’ package v. 7.3-60.0.1 [61]. We used the ‘emmeans’ package for pairwise comparisons [56]. Then, in additional models examining the relationship between relative timing and breeding success (excluding 2013 and 2014 data when individual ages were not tracked), we included parental age and the interaction between age and relative timing. In these models, we were specifically interested in whether parental age influenced the relationship between relative timing and breeding success. Separate models were run for males and females. If the interaction between relative timing and age of either parent was significant, models were run for each age group separately.

### 2.4. Continuous timing analysis

For each year of breeding data, and separately for each elevation, we calculated the relative timing of breeding for each nest by subtracting the earliest recorded first egg date from the first egg date of each nest. Thus, a nest that started 7 days after the earliest nest of that year was given a relative timing value of 7. We first modelled the effect of relative timing on breeding output and success using linear models with year as a fixed effect and an interaction between year and relative timing. We also modelled the effect of relative timing with a quadratic fit using the ‘poly’ function from the ‘stats’ package v. 3.6.2 (orthogonal polynomials [55]) to test for patterns of lower reproductive success in early and late nests. If the relative timing terms were significant in both models with a linear fit and models with a quadratic fit, we then compared the models using the ‘anova’ function from the ‘stats’ package to determine whether a quadratic or linear fit best described the relationships between relative timing and the reproductive parameters [55]. Type III ANOVA test results for models with linear and quadratic terms are included in the Supplementary Material. If the interaction between year and relative timing was significant, models were run for each year separately and linear and quadratic models were compared for each year as described above.

Next, we modelled the effect of parental age on relative timing with an interaction between age and elevation using linear models with a Gaussian distribution and used the ‘emmeans’ package for pairwise comparisons [56]. In additional models examining the relationship between relative timing and reproductive success, we included male and female ages (excluding 2013 and 2014 data) and the interaction between age and relative timing. In these models, year was included as a random effect. If the interaction between relative timing and age of either parent was significant, models were run separately for each age (first-time breeders or older breeders) and linear and quadratic models were compared as described above. The same R packages and methods described for the categorical timing analysis were used for modelling, simulating residuals and computing ANOVA tables.

## 3. Results

Our analyses included a total of 1090 nests: 453 nests at high (mean: 41 nests per year) and 637 nests at low elevation (mean: 58 nests per year). Of these nests, female ages were known for 353 nests at high elevation and 463 nests at low elevation, and male ages were known for 353 nests at high elevation and 465 nests at low elevation. Across years, high-elevation birds consistently bred later than low elevation birds, though the shapes of the distributions and degree of overlap in timing between elevations varied year to year (figure 1). The range of peak breeding dates across years (based on 3-day sum categorizations of nests) at high elevation was 14 days, while the range of peak dates at low elevation was 11 days (figure 1). The within-season ranges of first egg dates (lengths of breeding seasons) at low elevation were significantly longer than the ranges of first egg dates at high elevation (low-elevation mean range: 28.7 ± 5.0 days; high-elevation mean range: 23.4 ± 5.5 days; *β* = 7.27, *z* = 3.41, *p* < 0.001).

### 3.1. Categorical timing analysis

#### 3.1.1. Timing of breeding

**Figure 3.**
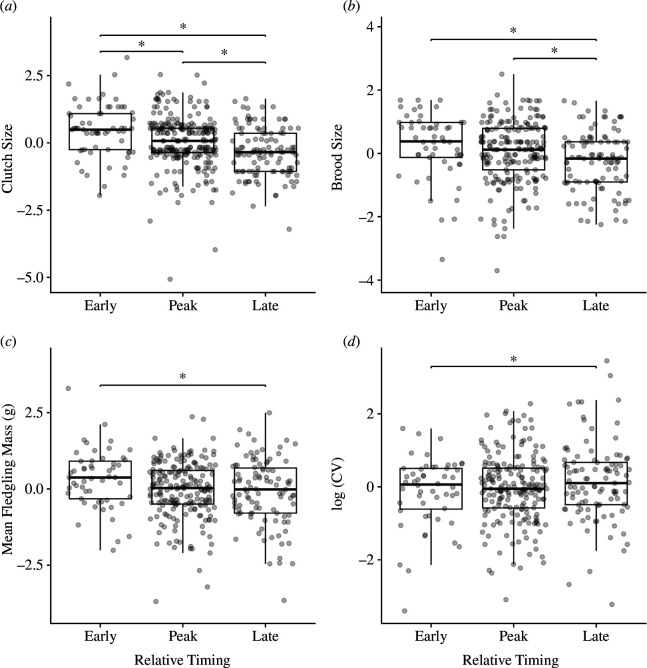
High elevation**:** relationships between categorical relative timing and breeding variables: (*a*) clutch size; (*b*) brood size; (*c*) mean fledgling mass; (*d*) log-transformed coefficient of variation in mean fledgling mass (CV). Response variables were centred within years for plotting. Asterisks (*) indicate significance of comparison (*ɑ* < 0.05). Data from 2013 to 2023.

**Figure 4.**
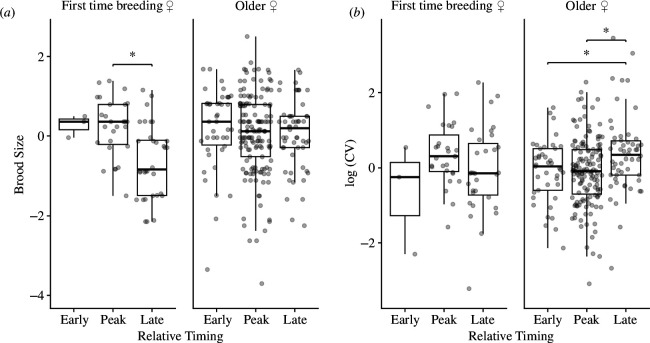
High elevation**:** interactions between categorical relative timing and parental age: (*a*) relationship between brood size and relative timing for the first-time breeding females and older females; and (*b*) relationship between within-nest CV in fledgling mass (log-transformed) and relative timing for the first-time breeding females and older females. Response variables were centred within years for plotting. Asterisks (*) indicate significance of comparison (*ɑ* < 0.05). Data from 2015 to 2023.

Mountain chickadees that bred later than the peak in nesting had lower breeding success at both low and high elevations (figures 3 and 4; tables A1 and A2)). Birds breeding during the peak in nesting never performed better than those breeding early, suggesting there was no clear fitness benefit to breeding at the peak versus breeding earlier.

At low elevation, there were no differences in clutch size between early and peak breeders, but both groups had larger clutches than late breeders (figure 2*a*, table A2). However, birds that bred late had smaller brood sizes (smaller number of fledglings) than birds that bred at the peak, but there were no differences between early and peak or early and late nests (figure 2*b*, table A2). There were no differences in mean fledgling mass among any of the groups at low elevation (figure 2*c*, table A2), but late nests were associated with larger variation in within-nest fledgling mass (CV) compared with early nests (figure 2*d*, table A2).

At high elevation, birds that bred early had the largest clutches, followed by those that bred at the peak and those that bred late (figure 3*a*, table A2). Both early and peak breeders had larger broods (larger number of fledglings) than late breeders, but brood size was not different between early and peak nests (figure 3*b*, table A2). Late nests were associated with smaller mean fledgling mass than early nests at high elevation, with no differences between early and peak nests (figure 3*c*, table A2). Similar to low elevation, at high elevation, late nests were also associated with larger variation in within-nest fledgling mass (CV) compared with early nests, with no difference between the other group pairs (figure 3*d*, table A2).

#### 3.1.2. Effects of parental age

At both elevations, older females bred earlier than first-time breeding females based on categorical relative timing (low elevation: *β* = 1.20, s.e. = 0.22, *z* = 5.57, *p* < 0.001; high elevation: *β* = 1.02, s.e. = 0.25, *z* = 4.10, *p* < 0.001), and there was no interaction between female age and elevation (table A3). However, male age was not a significant predictor of categorical relative timing (e.g. early-peak-late; *β* = −0.051, s.e. = 0.30, *z* = −0.17, *p* = 0.86), and there was no interaction between male age and elevation (*β* = −0.45, s.e. = 0.36, *z* = −1.26, *p* = 0.21; table A3).

There were no interactions between age and relative timing at low elevation (tables A4 and A5). However, parental age did influence relationships between timing and brood size and between timing and within-nest CV in fledgling mass, but only at high elevation (tables A4 and A5).

At high elevation, older females exhibited no relationship between relative timing and brood size, but for the first-time breeding females, peak nests were associated with larger mean fledgling mass compared with late nests (*β* = 0.22, *z* = 3.88, *p* < 0.001). There were no differences in mean fledgling mass between the early and peak (*β* = 0.060, *z* = 0.47, *p* = 0.88) or early and late nests (*β* = 0.28, *z* = 2.21, *p* = 0.07; Figure 4*a*).

In contrast, at high elevation, the relationship between relative timing and within-nest CV in fledgling mass was not significant for first-time breeding females, but in older females, late nests were associated with significantly larger within-nest CV in fledgling mass compared with early (*β* = 0.26, *z* = 2.76, *p* = 0.017) and peak nests (*β* = 0.26, *z* = 3.51, *p* = 0.002; Figure 4*b*). There was no difference in within-nest CV in fledgling mass between early and peak nests (*β* = 0.0048, *z* = 0.06, *p* = 1.00; Figure 4*b*).

### 3.2. Continuous timing analysis

#### 3.2.1. Timing of breeding

Models with only linear relative timing terms outperformed models with quadratic relative timing terms for most breeding parameters at both elevations (tables A6 and A7).

**Figure 5.**
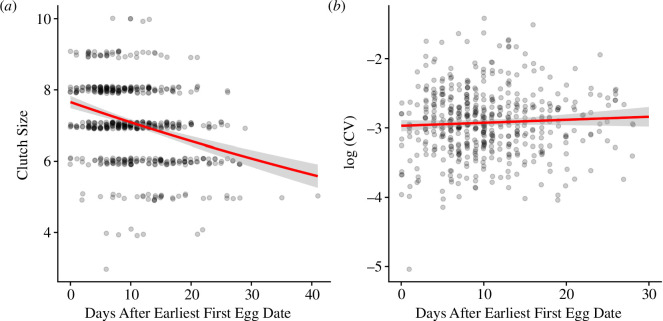
Low elevation**:** relationships between continuous relative timing (days after earliest first egg date) and breeding parameters: (*a*) clutch size; and (*b*) log-transformed coefficient of within-nest variation in mean fledgling mass (CV). Data from 2013 to 2023. Only significant relationships are shown.

**Figure 6.**
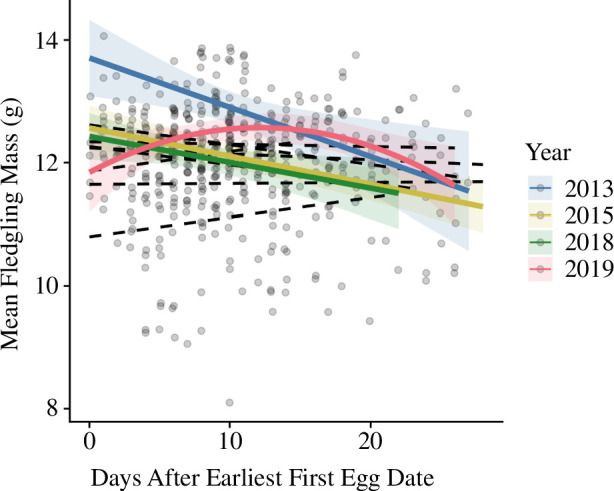
Low elevation**:** relationship between continuous relative timing (days after earliest first egg date) and mean fledgling mass. The interaction between year and relative timing was significant; thus, relationships are shown separately for each year. Years with significant relationships are shown with solid-coloured lines and years without significant relationships are shown with black dashed lines. Data from 2013 to 2023.

At low elevation, the relationship between clutch size and relative timing was best described by a linear fit, with clutch sizes decreasing with later breeding (figure 5*a*, table A6). However, brood size (number of fledglings) was not associated with relative timing at low elevation regardless of whether we used a linear or quadratic model fit (table A6). The relationship between mean fledgling mass and continuous relative timing was best described by a linear fit (table 7 A7), with later nests associated with lower mean fledgling mass (*β* = −0.081, *z* = −2.62, *p* = 0.009; Figure 5*b*, table A6), but there was also an interaction between relative timing and year (table A6). When each year was analysed separately, the relationships between mean fledgling mass and relative timing were best described by linear fits in 2013, 2015 and 2018, with lower mean mass in later nests (figure 6, table A8). However, in 2019, the relationship between mean mass and relative timing was best described by a quadratic relationship, with early and late nests associated with lower mean fledgling mass (figure 6, table A8). The relationship between within-nest CV in fledgling mass and relative timing was best described by a linear fit, with CV increasing in later nests (*β* = 0.052, *z* = 2.96, *p* = 0.003; Figure 5*b*, table A6).

**Figure 7.**
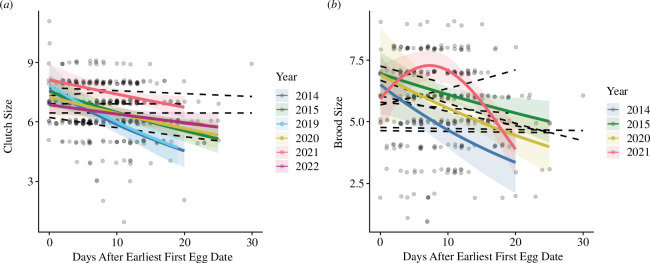
High elevation: relationships between continuous relative timing (days after earliest first egg date) and breeding parameters: (*a*) clutch size; and (*b*) brood size. The interaction between year and relative timing was significant; thus, relationships are shown separately for each year. Years with significant relationships are shown with solid-coloured lines and years without significant relationships are shown with black dashed lines. Data from 2013 to 2023.

At high elevation, the clutch size model with the quadratic term performed slightly better; however, the main effect of relative timing was not statistically significant (tables A6 and A7). There was an interaction between relative timing and year, and when the quadratic relationship was present, this relationship was the opposite of what we predicted (larger clutch sizes in early and late nests). When each year was analysed separately, the relationship between clutch size and relative timing was best described by linear, rather than quadratic, relationships between clutch size and timing, with later nests associated with smaller clutches in most years (figure 7*a*, table A9). In 2023, the relationship between clutch size and relative timing was best described by a quadratic model fit (table A9); however, visual analysis of model fit suggested that this relationship was driven by one data point, and when this point was removed, the relationship was no longer quadratic. Therefore, linear models are reported as the most meaningful models for the relationship between clutch size and relative timing at high elevation.

At high elevation, while brood size (number of fledglings) was not associated with relative timing itself, the interaction between relative timing and year was significant in the model with the quadratic term (table A6). When each year was analysed separately, the relationship between brood size and relative timing was best described by linear relationships in 2013, 2015 and 2018, with later nests associated with smaller broods (figure 7*b*, table A10). In contrast, in 2019, the relationship between brood size and relative timing was best described by a quadratic relationship, with early and late nests associated with smaller broods (figure 7, table A10). At high elevation, neither mean fledgling mass nor CV were associated with relative timing (table A6).

#### 3.2.2. Effects of parental age

**Figure 8.**
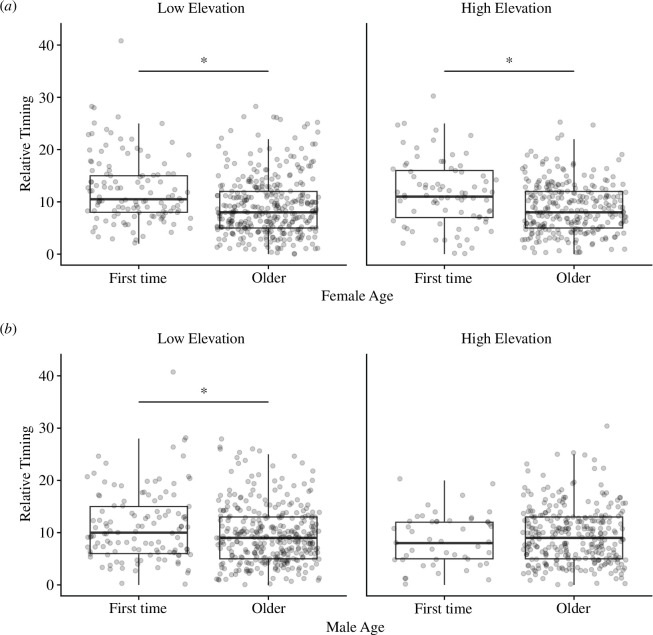
Relationships between (*a*) female and (*b*) male ages and continuous relative timing at low and high elevations.

At both elevations, older females bred earlier than first-time breeding females based on continuous relative timing (low elevation: *β* = 3.31, s.e. = 0.60, *t* = 5.55, *p* < 0.001; high elevation: *β* = 2.84, s.e. = 0.72, *t* = 3.97, *p* < 0.001), and there was no interaction between female age and elevation (figure 8, table A11). However, there was an interaction between male age and elevation, where older males bred earlier than first-time breeding males at low elevation (*β* = 1.82, s.e. = 0.60, *t* = 3.04, *p* = 0.013) but not at high elevation (*β* = −0.77, s.e. = 0.86, *t* = −0.89, *p* = 0.81; Figure 8, table A11).

When age was added to the models investigating the relationship between relative timing and breeding success, there were no interactions between parental age and continuous relative timing for any of the breeding parameters measured in this study (tables A12–A15).

## 4. Discussion

Our results suggest an overall fitness benefit to early breeding timing (before the peak of breeding) at high elevation (i.e. larger clutch size) and similar benefits to both early and peak breeding at low elevation. In contrast, later breeders had reduced reproductive success (i.e. smaller clutch size, fewer fledglings and lower fledgling mass) at both elevations. Furthermore, we found no clear benefit to breeding synchronously during the peak of breeding compared with breeding before the peak. These results are in general agreement with previous work in other systems showing a decline in fitness with later breeding [19,26,28,30]. In addition, older, experienced females bred earlier compared to first-time breeding females at both elevations, but this relationship was not as strong for males, suggesting that female age (associated with experience or other survival-related traits) is an important factor affecting breeding phenology.

When nests were categorized as ‘early’, ‘peak’ or ‘late’ based on the distributions of first egg dates within each year, early and peak nests both performed better than late nests, which had the lowest clutch sizes, the smallest number of fledglings and lowest mean fledgling masses at both elevations. At low elevation, early and peak nests performed equally well and better than late nests for clutch size, brood size and within-nest CV in fledgling mass. At high elevation, early nests were associated with larger clutches compared with the peak nests, but early and peak nests had similar numbers of fledglings, mean fledgling masses and CV. These results suggest that even though many birds synchronize their reproduction, breeding during the time when the majority of the population breeds may not result in fitness benefits over breeding early.

Overall, models with linear continuous relative timing terms performed better than models with quadratic terms, which added further support to the results from the categorical analysis demonstrating no clear advantage to breeding synchronously at the peak of the nesting distribution. At low elevation, clutch size and mean fledgling mass decreased linearly with relative timing, although the relationship between relative timing and mean fledgling mass differed among years. CV in fledgling mass increased linearly with relative timing at low elevation, such that fledglings from later nests varied more in their mass, suggesting that later nests struggled to invest equally in all nestlings. At high elevation, while continuous relative timing itself was not a significant predictor of any breeding parameters, the relationships between relative timing and clutch size and relative timing and brood size (number of fledglings) were significant in some years (clutch size: 2014, 2015 and 2019–2022; brood size: 2014, 2015, 2020 and 2021), with most of these years showing a linear relationship where clutch or brood size decreased with time. Notably, the 4 years in which brood size showed a significant relationship with relative timing at high elevation were low to moderate snow years (<200 cm maximum snow depth in the preceding winter, [45,46]). This may suggest that high snow years present a different set of environmental conditions that change the consequences of breeding timing (e.g. breeding early may be more difficult even for experienced individuals during heavy drought or snow years owing to overall low food abundance). Notably, we did not find evidence of a reverse trend where breeding success increased with time in any year (as in [11–14]) at either elevation, such that breeding late was never associated with higher fitness, even during extreme snow years (2017, 2019 and 2023).

Quadratic relationships between relative timing and breeding success were observed for mean fledgling mass at low elevation in a single year (2019) and brood size (number of fledglings) at high elevation in a single year (2021). This suggests that earlier breeding may have costs in certain years with specific environmental or ecological conditions. For example, the match–mismatch hypothesis may be more relevant in some years compared with others if particularly early breeders are not matching optimal food abundance. In fact, Vatka *et al*. [62] demonstrated that in boreal populations of great tits (*Parus major*) and willow tits (*Poecile montanus*), birds that better matched the food peak raised heavier young, but only during years with high caterpillar abundance. This suggests that annual differences in food abundance may affect the relationship between relative timing and breeding success, particularly in more northern latitudes or at higher elevations. Similarly, in our population, breeding success differs significantly across years [45,46], suggesting that variation in environmental variables such as overall food abundance across years may affect the overall breeding success of mountain chickadees, though we were not able to directly test this owing to lack of data on seasonal invertebrate abundance.

Resident birds in harsher climates may experience selection to breed earlier because early hatching is associated with early establishment and dominance in winter flocks and increased likelihood of overwinter survival in juveniles [20,21]. In northern latitudes and at higher elevations, the extended winter period constrains the breeding season such that birds have limited time to breed, molt and prepare for winter. In these systems, breeding early may be beneficial, especially in years when winter onset is earlier [63]. However, birds that breed early may be exposed to worse environmental conditions (e.g. low food abundance), at least in the beginning of the breeding period, resulting in a tradeoff (e.g. [64]). In our system, high elevation chickadees breed notably later than chickadees at low elevation owing to longer lasting snow cover (in some years, snow at high elevation lasts until mid-July; [45]), limiting time to breed. As a result, breeding as early as possible may be especially important at high elevation. However, overall patterns of timing and reproductive success were similar between elevations with later breeders having the lowest reproductive success.

In long-lived vertebrates, increased parental quality (more experienced individuals or individuals with traits allowing them to succeed under suboptimal conditions, etc.) is one factor that can affect the relationship between relative timing and breeding success. Individuals of high quality may be more capable of breeding earlier than would be expected based on food availability or other conditions. We expect that older birds are more experienced, allowing them to breed earlier and produce higher quality (more or larger) offspring even when breeding before the peak in food abundance [60]. In the current study, older birds also responded differentially to breeding early or late, such that older late breeders experienced decreased breeding success, while first-time breeders showed no difference in breeding success with relative timing. In these cases, older birds that breed late may be lower quality individuals. Alternatively, we may not have detected differences in breeding success across timing categories for first-time breeders simply owing to low sample sizes in the early breeding category, as first-time breeders were much less likely to breed early than older breeders.

Why is breeding late associated with reduced fitness? There are two hypotheses: environmental conditions could deteriorate within the season so later breeders experience worse conditions (e.g. food availability), and/or late breeders could simply be of lower quality [22]. Results from the selection experiment by Lindner *et al*. [39] may suggest that in wild populations, individual quality and not environmental differences is the main driver leading to decreased reproductive success observed later in the season. Lindner *et al*. [39] artificially selected birds for early or late breeding timing in captivity and found no difference in breeding success between the early and late breeding genetic lines. In this experiment, birds differed in their genotypes but likely did not differ in their quality (as the genetic lines were created in the same captive conditions). The lack of variation in individual quality may explain why Lindner *et al*. [39] reported no differences in breeding success despite genetic differences in breeding timing. Furthermore, some studies from boreal forests with high seasonality have shown that birds bred well before the food peak, suggesting that other factors beyond matching with peak food abundance are responsible for the patterns of reproductive timing and success in harsher climates [36,65,66]. In our system, we cannot definitively establish the drivers of reduced breeding success in late breeders, but it is likely that several different factors are responsible for within-season patterns of reproductive success.

An alternative explanation for low breeding success in late nests could be the occurrence of renests or second nesting attempts, which would be expected to have smaller clutches and broods. We have documented very few renests and second nesting attempts (~3% of all documented nests); however, it is possible that some of the extremely late nests included in our analyses could have been unidentified renests, though it is unlikely. We track individual birds with unique combinations of colour bands and PIT tags, which provides high confidence in recognizing late nests or renests in our nest boxes. However, for birds that initially nest in natural cavities and then renest in a nest box, it would be difficult to determine whether the nest is an initial or renest attempt. While it is possible that some of the late nests may be renests or second nesting attempts, it is unlikely that there were enough misclassified late nests to heavily skew the results. Furthermore, our results agree with other studies which also showed that late nests performed the worst [19,26,28,30].

## 5. Conclusions

Our results add support to many previous studies showing reduced breeding success in later breeding attempts [23–32]. Our study shows that even though the distribution of breeding activity within a season has a clear peak when most of the birds in the population breed in synchrony, there appear to be no clear fitness benefits to breeding during this peak, individuals breeding earlier performed as well or better than those breeding during the peak. At the same time, older, experienced parents, especially females, bred consistently earlier than the first-time breeders at both elevations. There appeared to be a fitness disadvantage to breeding later at both elevations despite large environmental differences between these elevations. Birds that bred late raised fewer fledglings that consistently weighed less than those from early or peak nests, though this may be owing to differences in individual parental quality if lower quality individuals bred late. It is likely that early breeders, especially at high elevations with harsher environments and shorter breeding seasons, may gain additional benefits over peak breeders in the form of increased fledgling recruitment (e.g. [20,21]). Considering such benefits of early breeding particularly in harsh environments, there may be strong fitness advantages to breeding as early as possible if an individual’s condition allows for successful reproduction. Future work should focus on testing whether these patterns in reproductive timing and success are related to seasonal and yearly variation in food supply.

## Figures and Tables

**Figure 9.**
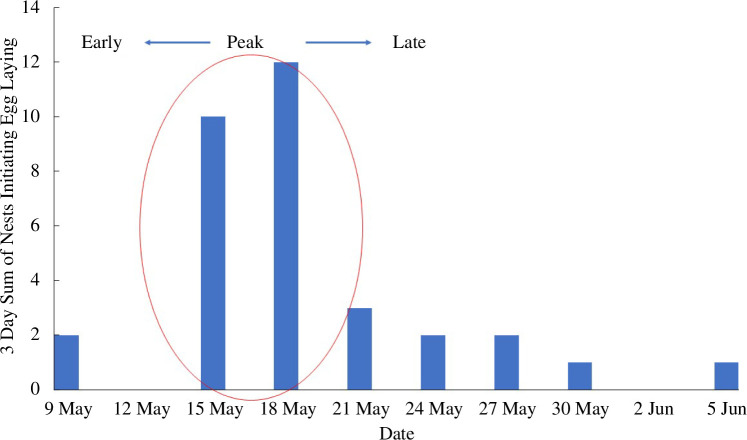
Illustration of how nests were categorized into early, peak and late nests for the categorical timing analysis. Data from low elevation in 2013.

## Data Availability

Data and code underlying the results of this study are available on Dryad [67].

## Appendix A

See figure 9 and tables 1 [Table T2] [Table T3] [Table T4] [Table T5] [Table T6] [Table T7] [Table T8] [Table T9] [Table T10] [Table T11] [Table T12] [Table T13] [Table T14]–15.

**Table 1.** Type II Wald *χ* ^2^ test results for models testing relationships between relative timing and clutch size, brood size, mean fledgling mass and log-transformed coefficient of within-nest variation in fledgling mass (CV), with year as a random effect. These models do not include male and female ages, and thus include all years of data from 2013 to 2023. Bolded *p*-values indicate significance (*ɑ* < 0.05).

		low elevation			high elevation		
response variable	predictor variable	*χ* ^2^	d.f.	*p*‐value	*χ* ^2^	d.f.	*p*‐value
clutch size	relative timing	34.17	2	**<0.001**	39.69	2	**<0.001**
brood size	relative timing	14.27	2	**<0.001**	14.34	2	**<0.001**
mean mass	relative timing	5.68	2	0.058	7.32	2	**0.026**
	brood size	2.69	1	0.10	0.023	1	0.88
CV	relative timing	7.62	2	**0.022**	6.85	2	**0.033**
	brood size	19.88	1	**<0.001**	2.96	1	0.085

**Table 2. T2:** Pairwise comparisons between early, peak and late nests for simple models testing relationships between categorical relative timing and breeding parameters without age at low elevation. The coefficient of within-nest variation in fledgling mass (CV) was log-transformed for analysis. Bolded *p* values indicate significance (*ɑ* < 0.05).

	*Early–peak*			Early–late			Peak–late		
	*β*	*z*	*p‐*value	*β*	z–	*p*‐value	*β*	*z*	*p*‐value
*low elevation*									
clutch size	0.029	1.71	0.20	0.080	4.98	**<0.001**	0.059	4.84	**<0.001**
brood size	−0.005	−0.18	0.98	0.068	2.29	0.057	0.073	3.68	**<0.001**
CV	−0.11	−1.79	0.18	−0.18	−2.73	**0.018**	−0.069	−1.52	0.28
*high elevation*									
clutch size	0.095	4.02	**<0.001**	0.17	6.28	**<0.001**	0.076	3.80	**<0.001**
brood size	0.047	1.19	0.46	0.15	3.40	**0.002**	0.11	3.20	**0.004**
mean mass	0.28	2.35	0.051	0.35	2.62	**0.025**	0.072	0.76	0.73
CV	−0.086	−1.13	0.50	−0.21	−2.45	**0.039**	−0.12	−2.01	0.11

**Table 3. T3:** Type III Wald *χ* ^2^ test results for ordinal regression testing relationships between (A) female and (B) male ages and categorical relative timing, with an interaction between age and elevation. Bolded values indicate significance (*ɑ* < 0.05).

predictor	*χ* ^2^	d.f.	*p*‐value
*(A) female age*			
age	16.92	1	**<0.001**
elevation	0.41	1	0.52
age:elevation	0.032	1	0.86
*(B) male age*			
age	0.030	1	0.86
elevation	3.79	1	0.052
age:elevation	1.58	1	0.21

**Table 4. T4:** Type III Wald *χ* ^2^ test results for models testing relationships between categorical relative timing and breeding parameters, including male age, the interaction between age and relative timing and the random effect of year. The coefficient of within-nest variation in fledgling mass (CV) was log-transformed for analysis. Data from 2015 to 2023. Bolded values indicate significance (*ɑ* < 0.05).

		low elevation			high elevation		
variable	predictor	*χ* ^2^	d.f.	*p*‐value	*χ* ^2^	d.f.	*p*‐value
clutch	relative timing	15.46	2	**<0.001**	2.06	2	0.36
	male age	2.12	1	0.15	1.06	1	0.30
	relative timing:male age	4.48	2	0.11	0.54	2	0.77
brood	relative timing	5.96	2	0.051	1.32	2	0.52
	male age	0.013	1	0.91	0.005	1	0.94
	relative timing:male age	0.49	2	0.78	0.99	2	0.61
mean mass	relative timing	1.80	2	0.41	2.62	2	0.27
	brood	2.36	1	0.12	0.42	1	0.52
	male age	3.58	1	0.058	0.50	1	0.48
	relative timing:male age	3.51	2	0.17	3.92	2	0.14
CV	relative timing	2.08	2	0.35	3.43	2	0.18
	brood	12.52	1	**<0.001**	3.56	1	0.059
	male age	3.58	1	0.058	0.86	1	0.35
	relative timing:male age	3.40	2	0.18	0.96	2	0.62

**Table 5. T5:** Type III Wald *χ* ^2^ test results for models testing relationships between categorical relative timing and breeding parameters, including female age, the interaction between age and relative timing and the random effect of year. The coefficient of within-nest variation in fledgling mass (CV) was log-transformed for analysis. Data from 2015 to 2023. Bolded values indicate significance (*ɑ* < 0.05).

		low elevation			high elevation		
variable	predictor	*χ* ^2^	d.f.	*p*‐value	*χ* ^2^	d.f.	*p*‐value
clutch	relative timing	10.16	2	**0.006**	6.90	2	**0.032**
	female age	0.37	1	0.54	3.01	1	0.083
	relative timing:female age	2.37	2	0.31	1.89	2	0.39
brood	relative timing	2.54	2	0.28	12.43	2	**0.002**
	female age	0.58	1	0.45	0.0002	1	0.99
	relative timing:female age	0.06	2	0.97	6.57	2	**0.037**
mean mass	relative timing	0.008	2	1.00	0.13	2	0.94
	brood	1.37	1	0.24	0.084	1	0.77
	female age	0.25	1	0.62	0.75	1	0.39
	relative timing:female age	0.11	2	0.95	1.01	2	0.60
CV	relative timing	0.30	2	0.86	5.07	2	0.079
	brood	16.58	1	**<0.001**	0.09	1	0.76
	female age	0.0005	1	0.98	1.22	1	0.27
	relative timing:female age	0.41	2	0.81	11.34	2	**0.003**

**Table 6. T6:** Type III Wald *χ* ^2^ test results for models with linear continuous relative timing terms only and models with quadratic relative timing terms. Models include year as a fixed effect and the interaction between year and relative timing. The coefficient of within-nest variation in fledgling mass (CV) was log-transformed for analysis. Data from 2013 to 2023. Bolded *p*-values indicate significance (*ɑ* < 0.05).

		linear			quadratic^a^		
variable	predictor	*χ* ^2^	d.f.	*p*‐value	*χ* ^2^	d.f.	*p*‐value
*low elevation*							
clutch linear *R* ^2^: 0.040 quadratic *R* ^2^: 0.045	relative timing	4.89	1	**0.027**	5.18	2	0.075
	year	30.56	10	**<0.001**	88.33	10	**<0.001**
	relative timing:year	12.82	10	0.23	26.54	20	0.15
brood linear *R* ^2^: 0.041 quadratic *R* ^2^: 0.052	relative timing	0.13	1	0.71	1.44	2	0.49
	year	11.60	10	0.31	48.56	10	**<0.001**
	relative timing:year	5.88	10	0.83	20.01	20	0.46
mean mass linear *R* ^2^: 0.175 quadratic *R* ^2^: 0.186	relative timing	6.86	1	**0.009**	7.19	2	**0.028**
	year	76.03	10	**<0.001**	106.16	10	**<0.001**
	elative timing:year	19.21	10	**0.038**	33.10	20	**0.033**
	brood	3.25	1	0.071	3.76	1	0.052
CV linear *R* ^2^: 0.031 quadratic *R* ^2^: 0.061	relative timing	8.78	1	**0.003**	9.17	2	**0.010**
	year	28.47	10	**0.002**	26.09	10	**0.004**
	relative timing:year	15.39	10	0.12	26.94	20	0.14
	brood	22.14	1	**<0.001**	22.96	1	**<0.001**
*High elevation*							
clutch linear *R* ^2^: 0.055 quadratic *R* ^2^: 0.061	relative timing	0.22	1	0.64	1.29	2	0.53
	year	28.98	10	**0.001**	50.19	10	**<0.001**
	relative timing:year	34.93	10	**<0.001**	36.87	20	**0.012**
brood linear *R* ^2^: 0.096 quadratic *R* ^2^: 0.061	relative timing	1.32	1	0.25	2.18	2	0.34
	year	33.96	10	**0.002**	48.46	10	**<0.001**
	relative timing:year	18.17	10	0.052	33.32	20	**0.031**
mean mass linear *R* ^2^: 0.127 quadratic *R* ^2^: 0.141	relative timing	1.26	1	0.26	1.70	1	0.43
	year	22.84	10	**0.011**	34.14	10	**<0.001**
	relative timing:year	9.62	10	0.47	20.05	20	0.45
	brood	0.13	1	0.72	0.15	1	0.70
CV linear *R* ^2^: 0.020 quadratic *R* ^2^: 0.023	relative timing	0.47	1	0.49	1.56	2	0.46
	year	3.17	10	0.98	10.68	10	0.38
	relative timing:year	10.44	10	0.40	2.77	1	0.096
	brood	2.63	1	0.10	15.65	20	0.74

^a^ One *χ* ^2^ value is reported for the combination of quadratic and linear relative timing terms because the ‘poly’ function from the ‘stats’ package in *R* was used to denote the quadratic function, and only one *χ* ^2^ value is calculated by the Type III Wald *χ* ^2^ test. Because these terms were not significant, no further model results are reported.

**Table 7. T7:** ANOVA results comparing performance of linear versus quadratic relative timing models for the full 11-year dataset, 2013–2023. Models are only compared if relative timing terms were significant in both linear and quadratic models. The coefficient of within-nest variation in fledgling mass (CV) was log-transformed for analysis. Bolded *p* values indicate significance (*ɑ* < 0.05).

variable	AIC of linear	AIC of quadratic	*χ* ^2^	*p*‐value
*low elevation*				
mean mass	1205.4	1213.7	13.77	0.25
CV	637.6	646.9	12.66	0.32
*high elevation*				
clutch	1334.4	1336.0	20.42	**0.040**

**Table 8. T8:** Modelling results for the relationship between mean fledgling mass and continuous relative timing at low elevation when the data are separated by year. Models with linear terms only and models with quadratic terms are included for comparison. Bolded values indicate significance (*ɑ* < 0.05).

year	model	predictor	estimate	s.e.	Z value	*p*‐value
2013	linear	relative timing	−0.080	0.029	−2.83	**0.005**
	quadratic	relative timing	−2.56	0.97	−2.64	**0.008**
		relative timing^2^	−0.23	0.98	−0.24	0.81
2014	linear	relative timing	−0.032	0.028	−1.18	0.24
	quadratic	relative timing	−1.42	1.25	−1.14	0.26
		relative timing^2^	1.33	1.37	0.97	0.33
2015	linear	relative timing	−0.046	0.012	−3.73	**<0.001**
	quadratic	relative timing	−2.72	0.71	−3.84	**<0.001**
		relative timing^2^	−1.02	0.71	−1.43	0.15
2016	linear	relative timing	−0.0097	0.017	−0.57	0.57
	quadratic	relative timing	−0.39	0.70	−0.56	0.58
		relative timing^2^	−0.34	0.68	−0.49	0.62
2017	linear	relative timing	0.0016	0.022	0.070	0.94
	quadratic	relative timing	0.19	0.99	0.19	0.85
		relative timing^2^	1.11	1.10	1.01	0.31
2018	linear	relative timing	−0.042	0.020	−2.11	**0.035**
	quadratic	relative timing	−2.33	0.97	−2.40	**0.016**
		relative timing^2^	−1.32	1.18	−1.12	0.26
2019^a^	linear	relative timing	−0.012	0.014	−0.90	**0.37**
	quadratic	relative timing	−1.32	0.75	−1.78	0.076
		relative timing^2^	−2.14	0.91	−2.36	**0.019**
2020	linear	relative timing	0.032	0.030	1.04	0.30
	quadratic	relative timing	5.23	5.39	0.97	0.33
		relative timing^2^	2.93	4.61	0.63	0.53
2021	linear	relative timing	−0.030	0.016	−1.82	0.068
	quadratic	relative timing	−1.31	0.71	−1.85	0.065
		relative timing^2^	−0.20	0.70	−0.29	0.77
2022	linear	relative timing	−0.0039	0.023	−0.17	0.87
	quadratic	relative timing	−0.17	0.92	−0.18	0.86
		relative timing^2^	1.29	0.87	1.48	0.14
2023	linear	relative timing	0.032	0.034	0.95	0.34
	quadratic	relative timing	2.67	1.54	1.74	0.082
		relative timing^2^	2.42	1.68	1.44	0.15

^a^ Mean fledgling mass in 2019 is best described by a quadratic model (AIC of linear = 127.73, AIC of quadratic = 124.41, *χ* ^2^ = 5.32, *p* = 0.021).

**Table 9. T9:** Modelling results for the relationship between clutch size and continuous relative timing at high elevation when the data are separated by year. Models with linear terms only and models with quadratic terms are included for comparison. Bolded values indicate significance (*ɑ* < 0.05).

year	model	predictor	estimate	s.e.	z value	*p*‐value
2013	linear	relative timing	−0.002	0.003	−0.60	0.55
	quadratic	relative timing	−0.086	0.13	−0.66	0.51
		relative timing^2^	0.16	0.13	1.25	0.21
2014	linear	relative timing	−0.026	0.006	−4.14	<0.001
	quadratic	relative timing	−0.73	0.20	−3.74	<0.001
		relative timing^2^	0.015	0.23	0.06	0.95
2015	linear	relative timing	−0.015	0.005	−3.30	<0.001
	quadratic	relative timing	−0.52	0.16	−3.29	0.001
		relative timing^2^	0.006	0.15	0.04	0.97
2016	linear	relative timing	0.003	0.011	0.25	0.80
	quadratic	relative timing	0.025	0.11	0.23	0.82
		relative timing^2^	−0.15	0.11	−1.39	0.17
2017	linear	relative timing	−0.008	0.006	−1.40	0.16
	quadratic	relative timing	−0.26	0.18	−1.47	0.14
		relative timing^2^	−0.071	0.16	−0.45	0.65
2018	linear	relative timing	−0.00006	0.0045	−0.01	0.99
	quadratic	relative timing	−0.006	0.15	−0.04	0.97
		relative timing^2^	0.087	0.15	0.59	0.56
2019	linear	relative timing	−0.027	0.0084	−3.20	0.0014
	quadratic	relative timing	−0.59	0.21	−2.89	0.004
		relative timing^2^	0.18	0.19	0.96	0.34
2020	linear	relative timing	−0.012	0.0054	−2.30	0.022
	quadratic	relative timing	−0.45	0.18	−2.46	0.014
		relative timing^2^	−0.20	0.19	−1.06	0.29
2021	linear	relative timing	−0.0094	0.0043	−2.20	0.028
	quadratic	relative timing	−0.29	0.13	−2.28	0.023
		relative timing^2^	−0.13	0.13	−0.99	0.32
2022	linear	relative timing	−0.0071	0.0035	−2.03	0.042
	quadratic	relative timing	−0.29	0.15	−1.97	0.049
		relative timing^2^	0.22	0.14	1.54	0.12
2023^a^	linear	relative timing	−0.00001	0.005	0.00	1.00
	quadratic	relative timing	−0.085	0.20	−0.43	0.67
		relative timing^2^	0.56	0.20	2.80	0.005

^a^ Clutch size in 2023 is best described by a quadratic model (AIC of linear = 146.11, AIC of quadratic = 140.5; *χ* ^2^ = 7.66, *p* = 0.0056); however, this relationship is driven by one point and when the point is removed from the dataset, neither quadratic or linear relationships are significant.

**Table 10. T10:** Modelling results for the relationship between brood size and continuous relative timing at high elevation when the data are separated by year. Models with linear terms only and models with quadratic terms are included for comparison. Bolded values indicate significance (*ɑ* < 0.05).

year	model	predictor	estimate	s.e.	z value	*p*‐value
2013	linear	relative timing	−0.015	0.030	−0.50	0.62
	quadratic	relative timing	−7.38	9.64	−0.77	0.44
		relative timing^2^	−3.30	4.65	−0.71	0.48
2014	linear	relative timing	−0.033	0.013	−2.62	0.009
	quadratic	relative timing	−0.83	0.37	−2.26	0.024
		relative timing^2^	0.33	0.39	0.86	0.39
2015	linear	relative timing	−0.013	0.006	−2.37	0.018
	quadratic	relative timing	−0.44	0.18	−2.41	0.016
		relative timing^2^	−0.11	0.18	−0.60	0.55
2016	linear	relative timing	−0.011	0.020	−0.52	0.60
	quadratic	relative timing	−0.12	0.21	−0.59	0.55
		relative timing^2^	−0.12	0.21	−0.58	0.56
2017	linear	relative timing	−0.0007	0.008	−0.085	0.93
	quadratic	relative timing	−0.018	0.27	−0.067	0.95
		relative timing^2^	−0.007	0.24	−0.031	0.98
2018	linear	relative timing	0.011	0.006	1.73	0.084
	quadratic	relative timing	0.37	0.21	1.72	0.086
		relative timing^2^	0.035	0.21	0.17	0.87
2019	Linear	relative timing	−0.004	0.014	−0.30	0.76
	quadratic	relative timing	−0.086	0.32	−0.27	0.79
		relative timing^2^	−0.098	0.31	−0.32	0.75
2020	Linear	relative timing	−0.022	0.011	−2.07	0.038
	quadratic	relative timing	−0.68	0.37	−1.85	0.064
		relative timing^2^	−0.71	0.51	−1.41	0.16
2021^a^	linear	relative timing	−0.017	0.007	−2.36	0.019
	quadratic	relative timing	−0.65	0.21	−3.04	0.002
		relative timing^2^	−0.71	0.24	−3.02	0.003
2022	linear	relative timing	−0.011	0.007	1.52	0.13
	quadratic	relative timing	−0.45	0.29	−1.57	0.12
		relative timing^2^	−0.22	0.29	−0.75	0.46
2023	linear	relative timing	−0.001	0.008	−0.11	0.92
	quadratic	relative timing	−0.12	0.31	−0.37	0.71
		relative timing^2^	0.43	0.31	1.38	0.17

^a^ Brood size in 2021 is best described by a quadratic model (AIC of linear = 189.08, AIC of quadratic = 182.46; *χ* ^2^ = 8.62, p = 0.003).

**Table 11. T11:** Type III Wald *χ* ^2^ test results for the relationship between (A) female and (B) male age and relative timing, with an interaction between age and elevation. Bolded values indicate significance (*ɑ* < 0.05).

predictor	*χ* ^2^	d.f.	*p*‐value
*(A) female age*			
age	46.33	1	<0.001
elevation	1.23	1	0.27
age:elevation	0.26	1	0.61
(*B*) *male age*			
age	3.97	1	0.046
elevation	1.78	1	0.18
age:elevation	6.06	1	0.014

**Table 12. T12:** Type III Wald *χ* ^2^ test results for models with linear continuous relative timing terms only and models with quadratic relative timing terms at low elevation. Models include male age, the interaction between age and relative timing and year as a random effect. The coefficient of within-nest variation in fledgling mass (CV) was log-transformed for analysis. Data from 2015 to 2023. Bolded values indicate significance (*ɑ* < 0.05).

		linear			quadratic		
variable	predictor	*χ* ^2^	d.f.	*p*‐value	*χ* ^2^	d.f	*p*‐value
clutch	relative timing	24.04	1	<0.001	26.45	2	<0.001
	male age	0.012	1	0.91	9.47	1	0.002
	relative timing:male age	3.41	1	0.065	2.03	2	0.36
brood	relative timing	8.39	1	0.004	10.34	2	0.006
	male age	0.010	1	0.92	3.26	1	0.071
	relative timing:male age	1.05	1	0.30	1.05	2	0.59
man mass	relative timing	0.79	1	0.37	2.90	2	0.23
	brood	2.57	1	0.11	2.75	1	0.097
	male age	7.04	1	0.008	4.20	1	0.040
	relative timing:male age	3.64	1	0.056	5.51	2	0.063
CV	relative timing	0.004	1	0.95	0.51	2	0.77
	brood	12.86	1	<0.001	12.80	1	<0.001
	male age	1.11	1	0.29	0.25	1	0.62
	relative timing:male age	0.97	1	0.32	2.37	2	0.31

**Table 13. T13:** Type III Wald *χ* ^2^ test results for models with linear continuous relative timing terms only and models with quadratic relative timing terms at low elevation. Models include female age, the interaction between age and relative timing and year as a random effect. The coefficient of within-nest variation in fledgling mass (CV) was log-transformed for analysis. These models include only data from 2015 to 2023. Bolded values indicate significance (*ɑ* < 0.05).

		linear			quadratic		
variable	predictor	*χ* ^2^	d.f.	*p*‐value	*χ* ^2^	d.f.	*p*‐value
clutch	relative timing	21.69	1	<0.001	22.24	2	<0.001
	female age	0.003	1	0.95	7.32	1	0.007
	relative timing:female age	2.72	1	0.099	2.08	2	0.35
brood	relative timing	4.57	1	0.033	4.88	2	0.087
	female age	0.41	1	0.52	4.51	1	0.034
	relative timing:female age	0.17	1	0.68	0.22	2	0.90
mean mass	relative timing	0.002	1	0.97	0.55	2	0.76
	brood	1.76	1	0.18	2.09	1	0.15
	female age	2.17	1	0.14	0.70	1	0.40
	relative timing:female age	1.86	1	0.17	3.48	2	0.18
CV	relative timing	0.016	1	0.90	0.18	2	0.91
	brood	17.65	1	<0.001	17.37	1	<0.001
	female age	1.22	1	0.27	0.23	1	0.63
	relative timing:female age	0.92	1	0.34	0.75	2	0.69

**Table 14. T14:** Type III Wald *χ* ^2^ test results for models with linear continuous relative timing terms only and models with quadratic relative timing terms at high elevation. Models include male age, the interaction between age and relative timing and year as a random effect. The coefficient of within-nest variation in fledgling mass (CV) was log-transformed for analysis. These models include only data from 2015 to 2023. Bolded values indicate significance (*ɑ* < 0.05).

		linear			quadratic		
variable	*predictor*	*χ* ^*2*^	d.f.	*p*‐value	*χ* ^2^	d.f.	*p*‐value
clutch	relative timing	0.18	1	0.67	0.88	2	0.64
	male age	2.10	1	0.15	1.61	1	0.20
	relative timing:male age	0.68	1	0.41	1.55	2	0.46
brood	relative timing	0.60	1	0.44	2.35	2	0.31
	male age	0.25	1	0.62	2.96	1	0.085
	relative timing:male age	0.013	1	0.91	1.43	2	0.49
mean mass	relative timing	0.0001	1	0.99	0.67	2	0.72
	brood	0.25	1	0.62	0.21	1	0.64
	male age	1.73	1	0.19	0.36	1	0.55
	relative timing:male age	1.09	1	0.30	1.86	2	0.39
CV	relative timing	3.33	1	0.068	6.27	2	0.044
	brood	2.64	1	0.10	2.69	1	0.10
	male age	1.48	1	0.22	0.28	1	0.60
	relative timing:male age	1.85	1	0.17	5.66	2	0.059

**Table 15.** Type III Wald *χ* ^2^ test results for models with linear continuous relative timing terms only and models with quadratic relative timing terms at high elevation. Models include female age, the interaction between age and relative timing and year as a random effect. The coefficient of within-nest variation in fledgling mass (CV) was log-transformed for analysis. These models include only data from 2015 to 2023. Bolded values indicate significance (*ɑ* < 0.05).

		linear			quadratic		
variable	predictor	*χ* ^2^	d.f.	*p*‐value	*χ* ^2^	d.f.	*p*‐value
clutch	relative timing	0.006	1	0.94	8.29	2	0.016
	female age	11.45	1	<0.007	4.78	1	0.029
	relative timing:female age	7.87	1	0.005	2.90	2	0.23
brood	relative timing	6.65	1	0.010	7.21	2	0.027
	female age	0.54	1	0.46	0.51	1	0.47
	relative timing:female age	3.31	1	0.069	4.36	2	0.11
mean mass	relative timing	2.71	1	0.10	4.09	2	0.13
	brood	0.012	1	0.91	0.021	1	0.88
	female age	0.14	1	0.71	1.42	1	0.23
	relative timing:female age	0.025	1	0.87	0.49	2	0.78
CV	relative timing	0.34	1	0.56	0.44	2	0.80
	brood	0.15	1	0.70	0.36	1	0.55
	female age	0.37	1	0.54	0.017	1	0.90
	relative timing:female age	0.47	1	0.49	3.70	2	0.16
